# Paired Capture and FISH Detection of Individual Virions Enable Cell-Free Determination of Infectious Titers

**DOI:** 10.1021/acssensors.3c00239

**Published:** 2023-06-27

**Authors:** Yifang Liu, Jacob L. Potts, Dylan Bloch, Keqing Nian, Caroline A. McCormick, Oleksandra Fanari, Sara H. Rouhanifard

**Affiliations:** Department of Bioengineering, Northeastern University, Boston, Massachusetts 02115, United States

**Keywords:** infectious titer, virus quantification, smFISH, aptamer, spike antigen

## Abstract

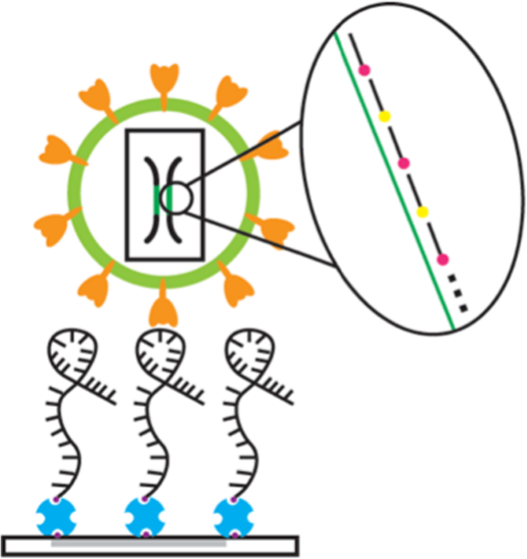

Early detection of viruses can prevent the uncontrolled spread of viral infections. Determination of viral infectivity is also critical for determining the dosage of gene therapies, including vector-based vaccines, CAR T-cell therapies, and CRISPR therapeutics. In both cases, for viral pathogens and viral vector delivery vehicles, fast and accurate measurement of infectious titers is desirable. The most common methods for virus detection are antigen-based (rapid but not sensitive) and polymerase chain reaction (PCR)-based (sensitive but not rapid). Current viral titration methods heavily rely on cultured cells, which introduces variability within labs and between labs. Thus, it is highly desirable to directly determine the infectious titer without using cells. Here, we report the development of a direct, fast, and sensitive assay for virus detection (dubbed rapid capture fluorescence in situ hybridization (FISH) or rapture FISH) and cell-free determination of infectious titers. Importantly, we demonstrate that the virions captured are “infectious,” thus serving as a more consistent proxy of infectious titers. This assay is unique because it first captures viruses bearing an intact coat protein using an aptamer and then detects genomes directly in individual virions using fluorescence in situ hybridization (FISH); thus, it is selective for infectious particles (i.e., positive for coat proteins and positive for genomes).

Rapid and accurate virus concentration measurement is desirable in both diagnostic and R&D settings. Clinically, rapid detection of infectious viral particles enables a timely diagnosis, and in industry, viral vectors are a significant component of gene therapies, vector-based vaccines, and CAR T immunotherapies. Currently, the most common methods for quantifying viruses are either based on the enzyme-linked immunosorbent assay (ELISA)-based detection of viral surface antigens,^1,2^ which is rapid but not sensitive, or polymerase chain reaction (PCR)-based detection of the viral genomes, which is sensitive but not rapid.^3,4^ A shortcoming of each of these assays is that the presence of either surface antigens or viral genomes does not necessarily indicate the concentration of infectious viral particles.

The infectious titer of a virus is often calculated in transducing units (TU) per mL. The total particle-to-TU ratio is the total number of particles divided by the TU. For many viruses, the particle-to-TU ratio can be extremely high and variable.^5^ For example, the ratio for the Varicella-zoster virus is 40,000:1,^6^ while for adenovirus (HAdV-C5), it is 58:1.^7^ For HIV-1, the ratios have even more variability ranging from 1 to 10^7^:1.^8^ In these cases, the biochemical properties measured of a viral sample may not correspond to the infectious titer of that sample, thus complicating the quality control and assessment of performance for gene therapies in vivo and evaluation of how infectious an individual is at a given moment.

The surface antigen test (ELISA) may detect viruses with surface antigens but no genome or free-floating antigens. By contrast, PCR-based tests may detect viruses that contain a genome but no surface antigens or RNA not encapsulated in a particle as well. For example, the viral RNA of SARS-CoV-2 patients can still be detected even in the absence of an infectious virus.^9^ Functional titering of infectious virions is accomplished with a viral plaque assay^10,11^ or tissue culture infectious dose-50 (TCID50),^12^ both cell-based. These methods titer virions by measuring the cytopathology in cell monolayers produced by viral replication. However, functional titering using cell culture can lead to significant variability^13^ due to cell type, passage number, and condition of the cells. Therefore, a cell-free titering method is needed and can improve the accuracy and consistency of viral quantification.

Single-molecule RNA fluorescence in situ hybridization (smFISH) is a sensitive method that directly detects RNA. It uses approximately 30 fluorescently labeled oligonucleotide probes to target RNA sequences.^14^ The tiling of probes across an RNA sequence amplifies the fluorescent signal locally and enables the direct detection of individual RNA molecules using fluorescence microscopy. SmFISH is frequently used to detect virus-infected cells^15−17^ and tissues.^18^ It has also been reported to detect individual virions via the capture of surface antigens;^19−21^ however, these methods required specialized instrumentation (i.e., Raman spectroscopy, TIRF microscopy). Two alternative smFISH methods, TurboFISH^16^ and rvFISH,^19−21^ can decrease the total time of the assay from 12 h to <20 min while maintaining the sensitivity and specificity of the assay, making these methods ideal candidates for rapid virus detection. However, TurboFISH detects viral genomes in infected cells, and rvFISH detects all viral particles containing the genome, irrespective of infectivity.

Here we report a rapid assay to directly detect intact virions (dubbed rapid-capture FISH or “rapture FISH”) using epifluorescence microscopy for targeting both viral genomes and coat protein, thus providing a better proxy for the overall infectivity of the detected virus. We apply both antibodies and aptamers that are specific for viral coat proteins to capture virions on the functionalized glass. Once captured, viral genomes were detected by TurboFISH to inform the infectivity of virions (positive for coat proteins and positive for genomes). The use of two probe sets targeting different genomic regions increases the specificity of viral RNA detection. We demonstrate that infectious virions are bound to the antibodies and aptamers by performing a functional titer on the unbound fraction of the virus, showing a marked decrease in infectivity. Overall, these results demonstrate that rapture FISH can be an effective complement to cell-based assays for determining infectivity, providing a useful and fast readout for manufacturers of gene therapies that are delivered via viral vectors, as well as a useful and fast measurement of the infectivity of an individual.

## Results

### Capture and Sandwich ELISA Detection of the Recombinant Spike Protein with an Anti-Spike Aptamer

**Figure 1:**
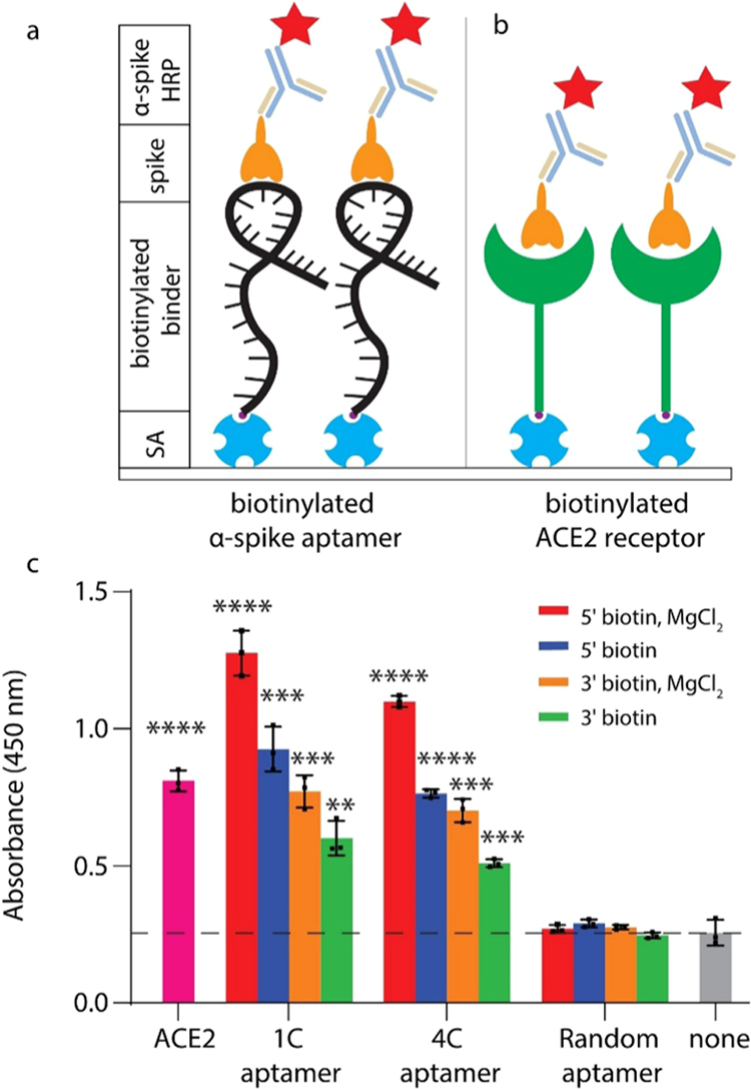
ELISA for capture efficiencies of anti-spike aptamers compared to the ACE2 receptor with the recombinant SARS-CoV-2 spike protein. (a) Aptamers modified with 5′ or 3′ biotin or (b) ACE2 proteins with biotin were conjugated to a streptavidin-coated polystyrene plate. Following spike binding (orange), the spike protein was detected with an anti-spike antibody conjugated to HRP, followed by colorimetric detection. (c) Measuring the binding specificity of 1C and 4C aptamers, different conditions of aptamers were tested (5′ modified, 3′ modified, ±0.55 mM MgCl_2_). *n* = 3 biological replicates (mean ± standard deviation (SD)). Plates without a biotinylated binder (none) were set as a baseline to show a significant difference from other groups. An unpaired *t*-test was performed to compare samples. *p < 0.05, **p < 0.01, ***p < 0.001, and ****p < 0.0001.

We selected previously reported DNA aptamer sequences (1C and 4C) that were computationally predicted to bind to the receptor-binding domain of the SARS-CoV-2 spike protein.^22^ The *K*_d_ values for these aptamers toward the spike trimer were reported as 5.8 and 19.9 nM, respectively.^22^ To evaluate the binding properties of the aptamers for our assay, we immobilized aptamers biotinylated on either the 5′ and 3′ ends to streptavidin-coated polystyrene plates (Figure 1a). As a positive control, we immobilized the biotinylated ACE2 protein to the streptavidin-coated polystyrene plates and used random aptamer sequences as our negative control (Figure 1b). We applied the recombinant SARS-CoV-2 spike protein (either in 1× phosphate-buffered saline (PBS) or in 1× PBS with 0.55 mM MgCl_2_) to the immobilized capture reagents and performed a sandwich ELISA using an horseradish peroxidase (HRP)-conjugated anti-spike antibody followed by colorimetric detection.^22−24^

We observed that the 1C aptamers had a stronger affinity toward the spike protein than the 4C aptamers. 5′ biotin-modified 1C had a 4.7-fold increase in absorbance, and the 5′ biotin-modified 4C aptamer had a 4.11-fold increase in absorbance as a random aptamer in the presence of MgCl_2_. 5′ biotin-modified aptamers captured 1.6-fold more spike protein than the 3′-modified aptamers. The presence of MgCl_2_ also had a positive effect on the quantity of the spike protein captured with a 1.44-fold increase in absorbance over the condition where MgCl_2_ was omitted, consistent with the previous work^22−24^ (Figure 1c). Overall, 5′ biotin-modified 1C aptamers exhibit the highest affinity toward the recombinant spike protein with MgCl_2_. This condition was used for the following experiments.

### Capture and Detection of Spike-Pseudotyped Lentiviruses with an Anti-Spike Aptamer Using RT-qPCR

**Figure 2:**
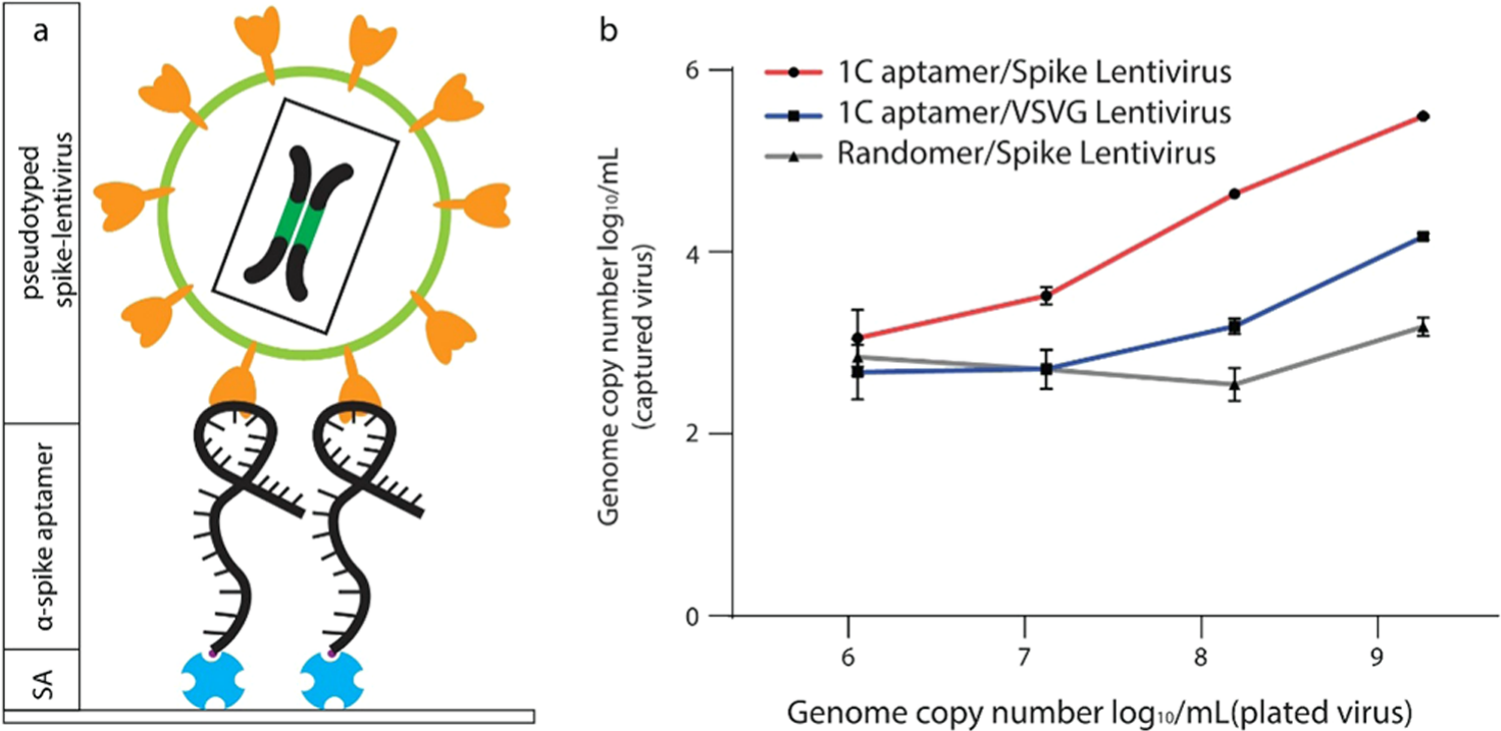
RT-qPCR for capture efficiencies of SARS-CoV-2 spike-pseudotyped lentivirus by 1C aptamers. (a) 1C aptamers modified with 5′ biotin were conjugated to a streptavidin-coated polystyrene plate. Following lentivirus capture by the aptamers, RNA was extracted, and RT-qPCR was performed to measure the number of captured virions. (b) Line graph of genome copy number (log_10_) per mL of spike and VSV-G virions captured by 1C aptamers and random aptamers. *n* = 3 biological replicates (mean ± SD).

To assess the binding efficiency of virions to the immobilized aptamers, we produced SARS-CoV-2 spike-pseudotyped lentiviral particles according to published work.^25^ We immobilized ACE2 and anti-spike aptamer 1C on streptavidin-coated polystyrene plates and exposed them to lentivirus virions to assess the capture efficiency. A random aptamer-coated plate and lentiviral particles pseudotyped with the vesicular stomatitis virus glycoprotein (VSV-G)^26^ were used as negative controls to assess the nonspecific binding of the virus to ACE2 and 1C aptamer. The spike-pseudotyped lentivirus and the VSV-G-pseudotyped lentivirus were added to the plates at concentrations ranging from 10^6^ to 10^9^ genome copies per mL (determined by RT-qPCR; Figure S1). After washing, we extracted the RNA from captured virions and performed RT-qPCR targeting the CMV promoter (common between both lentivirus vectors) to determine capture efficiency (Figure 2a).

Specific capture of spike-pseudotyped lentivirus was detected with the 1C capture reagent at a minimum concentration of 10^6^ genome copies per mL, showing a 2.4-fold increase in genome-containing units over the nonspecific virus captured by the random aptamer. When the concentration was increased to 10^9^ genome-containing units per mL, the capture efficiency increased, showing a 208.3-fold increase in genome copies over the nonspecific virus captured by the random aptamer (Figure 2b). The same capture efficiency was also observed in the ACE2 protein (Figure S2).

We then used a range of aptamer concentrations to coat the streptavidin plate to determine the optimal concentration for virion capture. The capture, RNA extraction, reverse transcription, and qPCR were performed, and six aptamer concentrations ranging from 1 to 200 μM were selected. We observed a 45.76-fold higher binding for the 5 μM aptamer concentration over the VSV-G virus and a 10.1-fold higher binding to 200 μM aptamer concentration. We observed that aptamer binding became saturated at around 10 μM (this concentration was used for all following experiments) and a 40-fold increase in spike-pseudotyped virus captured over the VSV-G virus at 10 μM (Figure S3).

### Rapture FISH Detection of Spike-Pseudotyped Lentiviruses with the 1C Aptamer

**Figure 3:**
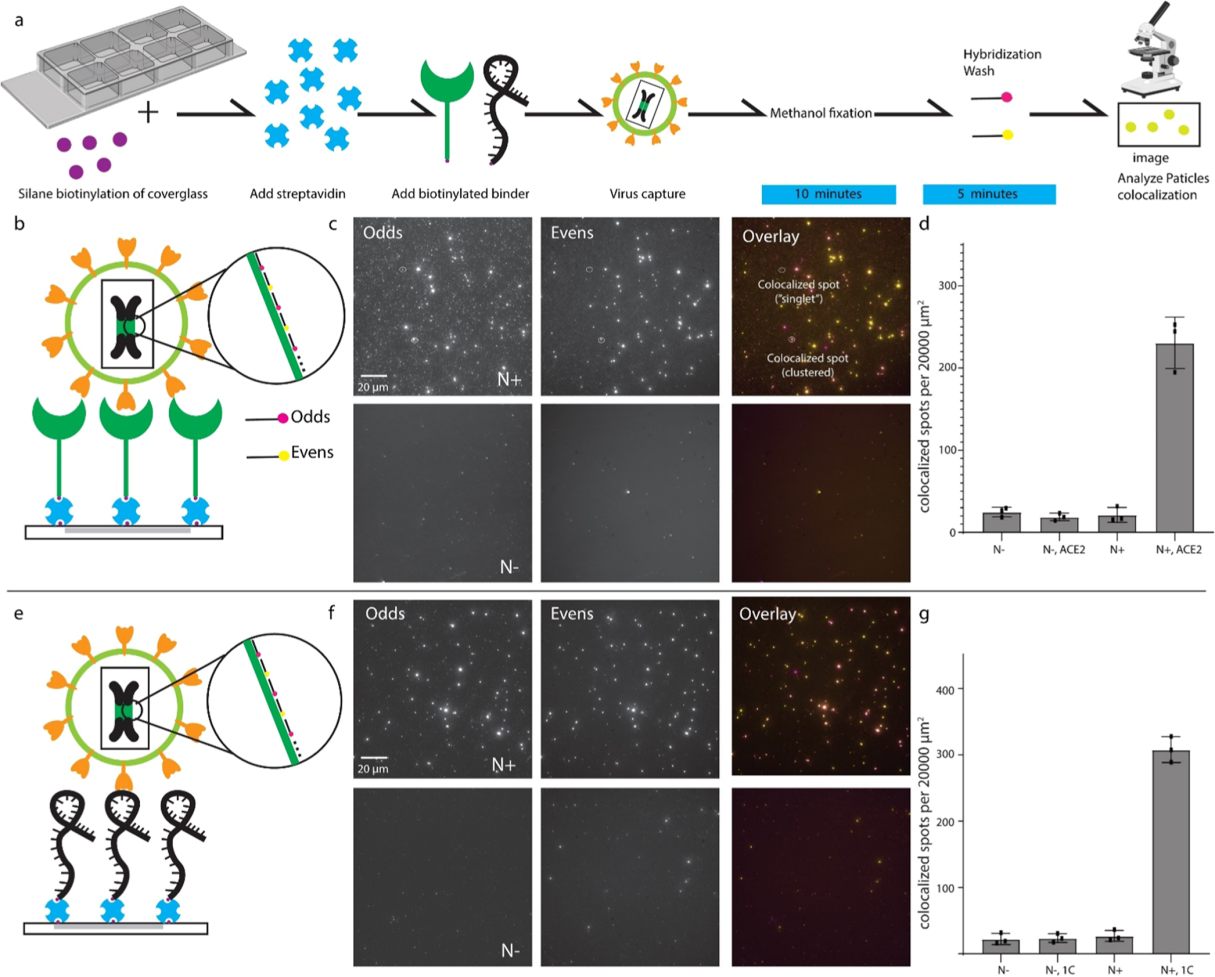
Rapture FISH detection of individual virions. (a) Workflow of rapture FISH detection. Rapture FISH using (b) ACE2 receptor or (e) 1C aptamers. Fluorescence micrographs showing rapture FISH targeting the N gene of the viral genome (c, f). (d, g) Colocalized FISH spots per 20,000 μm^2^ were quantified in bar graphs by the Matlab colocalization analysis pipeline. Three biological replicates, five images from each replicate (mean ± SD).

After validating that our system can capture viruses on polystyrene plates and chambered coverglass (Figure S4), we tested the utility of fluorescence in situ hybridization (FISH) to detect individual virions that are captured by aptamers in a method dubbed rapid-capture FISH (rapture FISH; Figure 3a). Previous work on detecting virions relies on smFISH, which employs hybridization times of up to 16 h;^19,27^ however, we applied an alternative method called TurboFISH^16^ that can decrease the hybridization time to 5 min to achieve fast detection. We confirmed that the virions would retain their integrity after fixation and permeabilization by capturing the virus and then performing RT-PCR on the captured virions post-methanol fixation and post-TurboFISH (Figure S5). The chambered coverglass was biotinylated by silane-biotin, and virions were captured as previously described. Briefly, the virus was fixed with methanol after specific capture, and smFISH probes^14^ targeting the SARS-CoV-2 nucleocapsid gene (N gene) were introduced and hybridized. After hybridization, there were further wash steps followed by epifluorescent microscopy (Figure 3a,b,e).

The size of the rapture FISH spots was determined to be 1–500 pixels by particle analysis in Matlab. Lentivirus is known to aggregate.^28^ We observed that 79.66% of detected spots were 1–70 pixels in diameter, showing up only in the samples where the virus was added. Therefore, we reasoned that colocalized spots in the 1–70-pixel range are likely individual virions. We observed that 20.4% of spots were beyond 70 pixels and these large spots were probable viral aggregates (Figure S6). The large particles (spots more than 70 pixels) showed the same colocalization rates as the small particles, indicating that the viral aggregates were infectious (Figure S6).

To confirm the detection of a true viral particle, we used two sets of probes (odds and evens) to mitigate the detection of false positive spots.^14^ The specificity of probes for the N gene was demonstrated previously.^18^ To confirm specific viral detection, we applied rapture FISH to a series of negative controls, including the slides not treated with the ACE2 protein and the slides not treated with 1C aptamers. Additionally, we tested viruses whose genome contains the N gene (N+) or not (N–; Figure 3b–g).

For the functionalized slides treated with ACE2 or 1C aptamer and in the presence of N+ virus, we observed a 10.88- and 12.88-fold increase in colocalized spots, respectively, compared to the samples without binding agents and the N gene (Figure 3b–g).

### Simultaneous Rapture FISH Detection of Multiple Virus Strains

**Figure 4:**
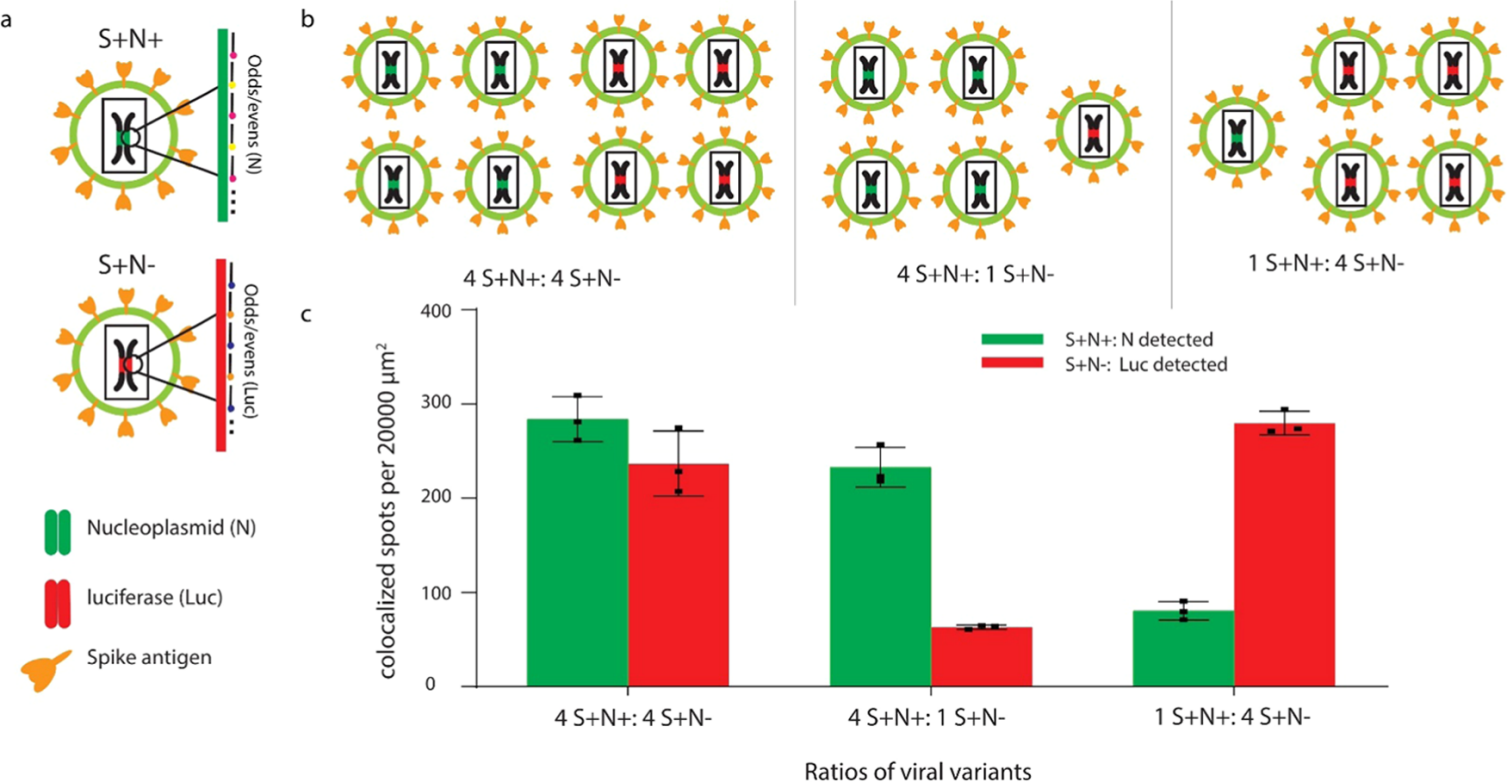
Multiplexed virus detection by TurboFISH. (a) Multiple virus strains with different genomes were detected by N gene probes and luciferase probes with two sets of dyes. (b) Different amount ratios of two virions were detected to show (c) whether the FISH spots ratio of two targets matches the concentration ratio of two virions added. Three biological replicates, five images from each replicate (mean ± SD).

To test whether the rapture FISH system can detect multiple strains, we performed the assay on a mixed population of spike-coated virions containing two distinct genomes. N+ virions, as described previously, were mixed in different ratios with virions containing the luciferase gene (N−). The N gene probe targeting the N gene of spike-coated lentivirus (S+N+) and the luciferase probe targeting the luciferase of spike-coated lentivirus without the N gene (S+N−) were combined to detect two types of virions simultaneously. Two sets of probes (odds and evens) with two dyes for each target were used to detect the colocalized FISH spots (Figure 4a). Virions were mixed with different concentration ratios, then captured, fixed, and detected by TurboFISH simultaneously (Figure 4b).

Three concentration ratios based on the RT-qPCR of the two virion types were used to test our system: 4:4, 1:4, and 4:1 of S+N+/S+N–. For the 4:4 ratio, the ratio of colocalized spots of S+N+ to S+N– was 1.2:1. For the 4:1 ratio, the ratio of colocalized spots of S+N+ to S+N– was 3.77:1. For the 1:4 ratio, the ratio of colocalized spots of S+N+ to S+N– was 1:3.55, consistent with the concentration ratio of the two virions we added (Figure 4c), indicating that rapture FISH can simultaneously detect multiple virion types.

### Functional Titering of the Remaining Supernatant from Virions Captured by Aptamers and ACE2 Receptors

**Figure 5:**
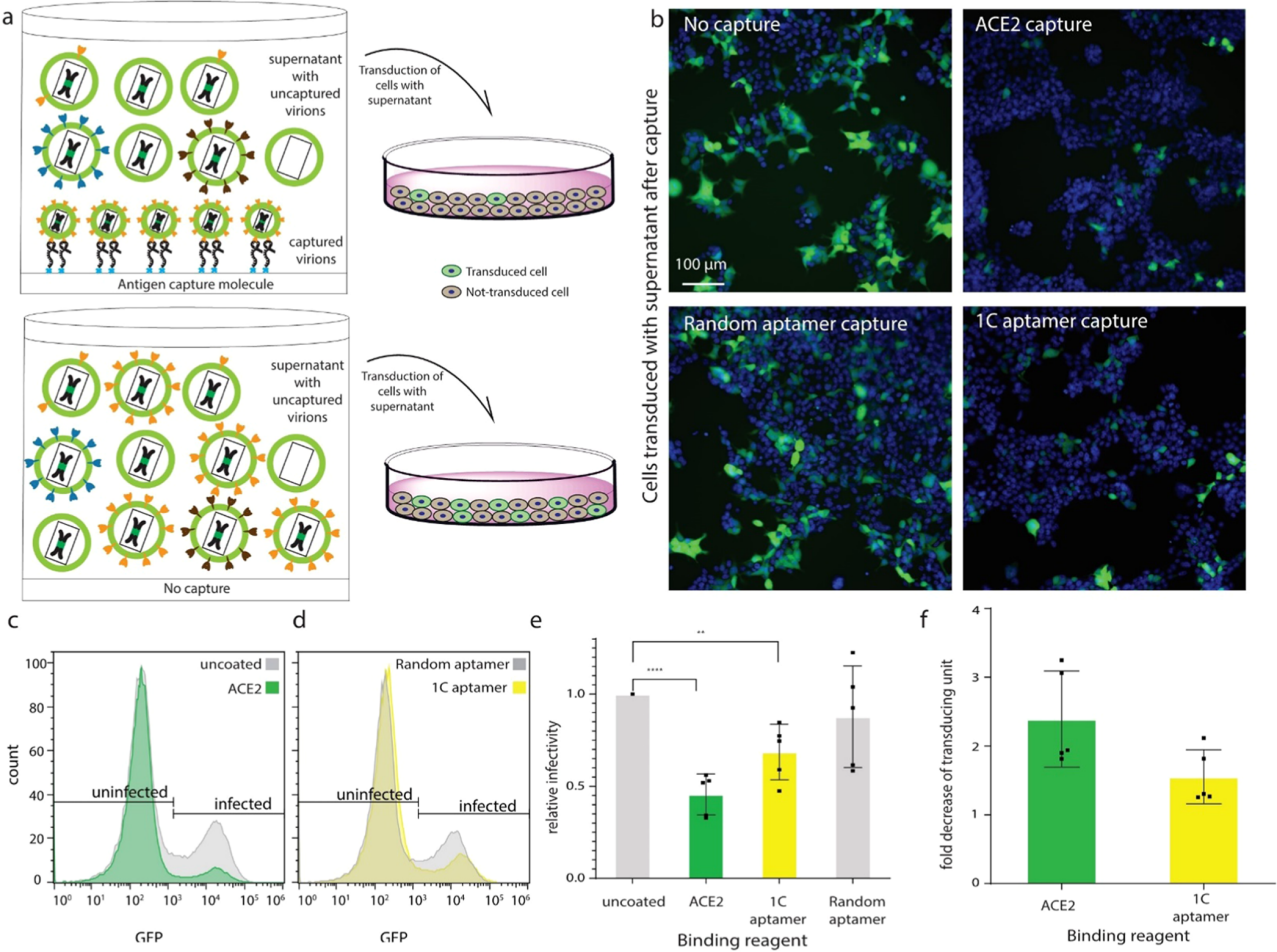
Functional titering of uncaptured virions from aptamers and ACE2 to prove the infectivity of captured virions. (a) Uncaptured supernatant virions were aspirated out and used to infect cells. Flow cytometry was performed to count the percentage of infected cells. (b) Images of cells infected by uncaptured virions from different binding reagents. Green is ZsGreen expression indicating viral infection. The cell nucleus was stained with 4′,6-diamidino-2-phenylindole (DAPI). (c, d) Histogram plots of flow cytometry data; uninfected/infected cells were gated based on viral infection of 293FT cells. The ACE2 group was compared to the uncoated group, and the 1C aptamer group was compared to the random aptamer group. (e) Normalization of uncaptured virions’ infectivity; the percentage of infected cells in different binding reagents was normalized to the uncoated group. (f) The fold decrease of TU/mL of ACE2 and 1C aptamer groups was compared to that of the uncoated group. *n* = 5 biological replicates (mean ± SD). *p < 0.05, **p < 0.01, ***p < 0.001, ****p < 0.0001.

Infectious titering relies on cell-based assays. Still, the accuracy and consistency of such methods heavily depend on cell conditions.^13^ Our system achieves a cell-free proxy for infectious titers. To test whether the infectious viral particles are indeed getting captured, we assessed the infectivity of the supernatant after incubating the virus on the ACE2 receptor and the 1C aptamer. The virion capture steps of aptamers and ACE2 were performed, as described in Figure 2. The uncaptured virions in the supernatant were aspirated and used to transduce ACE2-expressing HEK293T cells (HEK293T-ACE2). After incubating HEK293T-ACE2 cells with the virus for two days, the percentage of infected cells was measured with flow cytometry via ZsGreen expression from the viral genome (Figure 5a). Wild-type HEK293FT cells were infected simultaneously to set up the gate of uninfected cells. Plates not coated with aptamers (uncoated) were used to do normalization and measure the total amount of infectious particles. The percentage of infected cells was used to quantify the infectivity of different samples. Random aptamers were used as a negative control.

The percentage of infected cells decreased significantly after virion capture by ACE2 and 1C aptamers compared to the infectivity of viruses that were not captured, suggesting that the infectious particles are being captured. Random aptamers had similar infectivity as the no-capture group, which indicated that such capture is highly specific (Figures 5b and S7). Based on the flow cytometry data, two populations of uninfected/infected cells were gated in the histogram (Figure 5c,d). There was a 65.88% decrease in the percent of infected cells in the ACE2 group compared to the uncoated group (Figure 5c) and a 36.99% decrease in the percent of infected cells in the 1C aptamers group compared to the random aptamers group (Figure 5d). The random aptamer capture was comparable to the nonspecific capture on uncoated plates. The percentages of infected cells in ACE2, 1C, and random aptamers were normalized to that of the uncoated group (Figure 5e). The percentages of infected cells were transformed to TU/mL using the viral quantification formula.^25^ A 2.44-fold reduction and a 1.66-fold reduction in TU/mL were observed in the ACE2 group and the 1C aptamer group, respectively, compared to the uncoated group (Figure 5f).

### Relationship between Rapture FISH Detection of Infectious Virions and the Functional Viral Titer

**Figure 6:**
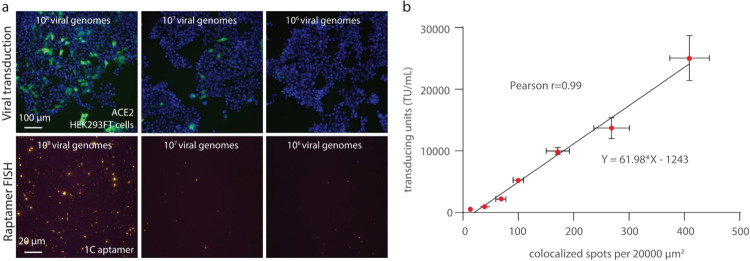
Correlation of rapture FISH detection with functional titering. (a) Images of infected ACE2-expressing HEK293T cells (green is ZsGreen expression indicating viral infection; top), and images of colocalized FISH spots from different concentrations of virions (bottom). The cell nucleus was stained with DAPI. (b) Quantification of infectious virions by comparison of colocalized FISH spots (*x* axis) with cell transducing units (y*x* axis). Points are showing the mean of three biological replicates (mean ± SD).

To assess the quantitative accuracy of our cell-free viral detection system, we measured the functional virus titer and the corresponding rapture FISH quantification. HEK293T-ACE2 cells were transduced with spike-pseudotyped lentivirus with the N gene at seven concentrations ranging from 10^6^ to 10^8^ genome units (determined by RT-qPCR; Figure 6a). TU were calculated from the percentage of ZsGreen positive cells (Figure 5). The same virus concentration (genome units) was applied to glass slides with the immobilized 1C aptamer to capture the spike-pseudotyped lentivirus with the N gene, followed by detection of the N gene with rapture FISH (Figure 6a). The colocalized spots per 20,000 μm^2^ increased proportionally with the transducing units (TU/mL) for the same genome-containing units (Pearson *r* = 0.99; Figures 6b and S8). The colocalized FISH spots were saturated when the virus concentration was over 10^8^ genome units, indicating the upper limit of capture and detection. This was consistent with the functional titer results that when the virus concentration was above 10^8^ genome units, the ZsGreen positive was over 10%, which made the viral quantification inaccurate^25^ (Figure S9).

## Discussion

Here, we outline rapture FISH, a cell-free platform for the capture and direct detection of infectious virions using aptamers for specific capture of virions, and TurboFISH for rapid and specific detection of the viral genome. This system offers several advantages over current technologies: (1) The DNA aptamers are thermostable and inexpensive and can reduce batch-to-batch variability. (2) TurboFISH allows for direct and specific detection of virions using standard epifluorescent microscopy. (3) Not all viruses are infectious; in this system, detection requires the presence of a surface antigen and genome, thus providing a cell-free proxy for infectious titers. (4) Finally, our image processing selects for size (to exclude debris and virus aggregates) and probe colocalization for the specificity of detection and uses multiple probes to discriminate different viruses in the sample.

We tested our technology on spike-pseudotyped lentiviruses bearing either the SARS-CoV-2 nucleocapsid gene or the luciferase gene. Our platform demonstrated specific spike-containing virus capture using ACE2 and the 1C aptamer. Further, it can detect viral genomes in 15 min and discriminate between multiple genomes. Importantly, we demonstrated that ACE2 and 1C capture infectious virions as viral supernatants after incubation with these agents relative to negative controls. We also demonstrate a strong correlation (Pearson *r* = 0.99) between the functional titer and the functional virions captured and detected by rapture FISH. This suggests the possibility of calculating TU/mL from spots/μm^2^ using a correction factor of 61.98. These properties make the paired aptamer capture and FISH detection of virions a viable tool for the cell-free determination of infectious virions in viral supernatants.

RT-PCR is most frequently used for viral measurements due to its sensitivity, which can detect as little as 100 copies of viral RNA per mL of transport media.^29,30^ However, the measurement of genome-containing units fails to account for infectivity (i.e., a virus that contains a genome but the surface antigen is damaged or mutant and therefore cannot infect a cell^31^). The infectivity is important for diagnostics because a person may have noninfectious virions or unpackaged viral nucleic acids remaining in their body but still produce a positive test.^9^ Likewise, the ELISA-based methods detect only the presence of the surface antigen and do not inform on infectivity (i.e., virions may contain the surface antigen without genome^32,33^). Alternatively, our capture and detection system, although theoretically single-molecule resolution, is limited by the capture efficiency of the aptamer or the antibody and, thus, will have lower sensitivity. However, this system combines the benefits of RT-PCR for genome detection and ELISA for surface antigen detection to inform on infectivity without the use of cells.

A major strength of this system is that aptamers may be selected via SELEX^34,35^ for any surface antigen, including other aptamers that target the spike protein for SARS-CoV-2,^36^ and RNA FISH probes may be designed to target different viral strains.^20,27^ These properties will allow the system to be rapidly customized and applied to emerging pathogens and any desired viral vector without the need for fluorescent transgenes that are either not present in natural pathogens or not permitted in the minimum payload for gene therapies.^37,38^

In summary, we report the development of a direct, fast, and sensitive assay for virus detection and cell-free determination of infectious titers with no thermal requirement. This assay is unique because it first captures viruses bearing an intact coat protein using an aptamer and then detects genomes directly in individual virions using FISH, thus selecting for “infectious” particles. Importantly, we demonstrate that the virions captured are “infectious,” thus serving as a proxy of infectious titers. This measurement will circumvent the need for cell-based assays to determine infectious titers for diagnostic and biotechnology settings.

## Methods

### Tissue Culture Conditions

HEK293FT cells (Thermo Fisher cat# R700-07) were handled according to the manufacturer’s protocol and maintained in high glucose Dulbecco’s modified Eagle’s medium (DMEM) with GlutaMax (Fisher Scientific cat# 10566016), 10% fetal bovine serum (FBS), 1% MEM non-essential amino acids (Thermo Fisher cat# R70007), 1% supplementary GlutaMax (Fisher Scientific cat# 35-050-061), 1% MEM sodium pyruvate (Thermo Fisher cat# 11360070), 1% Pen–Strep (Fisher Scientific cat# BW17-602E), and 500 μg/mL geneticin (Thermo Fisher cat# 10131035). HEK293T-ACE2 cells described previously^25^ (BEI cat# NR-52511) were cultured in the HEK293FT media without geneticin. Cell lines were tested PCR-negative for mycoplasma contamination (ATCC cat# 30-1012K).

### Plasmids

The following plasmids were ordered as part of a lentiviral kit (BEI cat# NR-52948) described previously:^25^ Spike (BEI cat# NR-52514), Luc2-ZsGreen (BEI cat# NR-52516), Hgpm2 (BEI cat# NR-52517), Tat1b (BEI cat# NR-52518), and Rev1b (BEI cat# NR-52519). The VSV-G plasmid (Addgene cat# 12259) was a gift from the lab of Jiahe Li. To prepare the spike G614 Δ19 plasmid, we generated two PCR amplicons using mutagenic primers, Q5 polymerase (NEB cat# M0491), and spike plasmid (BEI cat# NR-52514) as a template. PCR reactions were digested in *Dpn*I, and the amplicons were joined via Gibson assembly (NEB cat# E2611). The coding sequences of generated plasmids were sequence-verified. To prepare the N gene-inserted plasmids (CoV2Ngene-Luc2-ZsGreen) to model SARS-CoV-2, we removed the 1131 bp segment of luciferase in Luc2-ZsGreen (BEI cat# NR-52516), and the N gene (1260 bp, IDT 2019-nCoV_N_Positive Control Plasmid cat#10006625) was amplified by mutagenic primers. The remaining part of Luc2-ZsGreen (BEI cat# NR-52516) and the amplified N gene were ligated via Gibson assembly (NEB cat# E2611). The coding sequences of generated plasmids were sequence-verified.

### Generating Pseudotyped Lentiviral Particles

The protocol follows the previously described one in the literature^25^ with several modifications. HEK293FT cells were seeded in media without geneticin at a density such that cells were 50% confluent the next day. 16–24 h after seeding, the cells were transfected with the lentiviral plasmids using BioT (Bioland cat# B01-01) according to the manufacturer’s protocol. The number of plasmids per transfection was at a 1:2/1:9/1:6 mass ratio for the lentiviral backbone (Luc2-ZsGreen or N-ZsGreen), helper plasmids (Hgpm2, Tat1b, and Rev1b), and viral entry protein (Spike G614 Δ19, or VSV-G), respectively. Media was changed 18 h post-transfection (hpt). The viral supernatant was collected at 60 hpt and kept and centrifuged at 500*g* for 10 min to clear cell debris. The cleared supernatant was immediately frozen at −80 °C in aliquots.

### Viral Quantification

Viruses were quantified via a previously described RT-qPCR method,^39^ where we only deviated by using different DNase I (Thermo Fisher #AM1907) digestion, reverse transcriptase (Fisher Scientific #18-080-044), and plasmid (Luc2-ZsGreen) for generating standard curves. A CFX96 Optics Module, C1000 Touch Thermal Cycler (BIO-RAD) was used. Finally, viruses were functionally titered via HEK293T-ACE2 infection as previously described,^25^ with the only deviation being that the HEK293T-ACE2 cells were cultured in HEK293FT media without geneticin. 48 h post infection, the cells were analyzed with an Attune NxT flow cytometer for fluorescence, indicating a successful viral infection. The transducing units of infectious virions were obtained from P (percentage of infective positive cells) based on the following formula
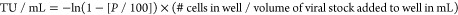


### Chemical Immobilization of Biotin to a Chambered Coverglass

To generate the biotinylated coverglass necessary to run and image rapture FISH, an eight-well chambered coverglass (Thermo Fisher #155409) was conditioned in an oxygen plasma cleaner to introduce the active oxygen group to the slide. The slide was cleaned with water and then with 100% isopropanol before the reaction. The slide was treated by oxygen at 300 mTorr pressure for 1 min and then by plasma power at 50 mW for 1 min. After the oxygen plasma, silane-PEG-biotin (10 mg/mL) dissolved in 99% ethanol was added to react with the oxygen group and incubated for 4–6 h at RT. The slide was washed with 99% ethanol three times after silane-PEG-biotin treatment. The dried slide was kept at 4 °C overnight for use the next day.

### ELISA Assay

The recombinant spike protein (BPS Bioscience #100810) was captured by the ACE2 receptor protein (Acro Biosystems #AC2-H82F9) and aptamers (CoV-2-RBD-1C and CoV-RBD-4C)-targeting receptor-binding domains of the SARS-CoV-2 spike glycoprotein, which were discovered in Song et al.^22^ Biotin-modified aptamers and ACE2 were bound to streptavidin-coated polystyrene plates (Thermo Fisher #PI15500). The aptamers and ACE2 captured the recombinant spike protein, and then SARS-CoV-2 spike protein monoclonal antibody [H6] HRP-conjugated (Assay Genie #PACOV0004) was used to detect the captured spike protein. The ELISA procedure to detect the spike protein was conducted according to the streptavidin-coated plate manufacturer’s protocol with the following changes: Aptamers (10 μM), spike protein (1 μg/mL), and monoclonal antibodies (1 μg/mL) were dissolved in the binding buffer (1× PBS, 0.55 mM MgCl_2_). The ACE2 protein (500 ng/mL) was dissolved in the wash buffer (25 mM Tris, 150 mM NaCl, 0.1% FBS, 0.05% Tween 20). The wells were prewashed with 1× PBS for 10 min and then with binding buffer for 10 min before adding the aptamers. The aptamers were heated at 90 °C for 10 min and placed on ice for 10 min. The aptamers and the ACE2 protein had 2 h of binding time. Both the spike protein and the antibody had 30 min of binding time. The plates were preincubated with HEK293T-ACE2 cell media with 0.55 mM MgCl_2_ for 10 min. The virus was prepared in HEK293T-ACE2 cell media with 0.55 mM MgCl_2_ and salmon sperm DNA. The virus was added to the plates and incubated for 45 min, and the plates were washed three times with binding buffer after virus incubation. For biotinylated coverglass, the streptavidin protein (100 μg/mL) was added first and incubated for 1 h, and the remaining steps were the same as described above.

### RNA Extraction, RT-PCR, and RT-qPCR

Methods were adapted from Zhang et al.^39^ Frozen viral aliquots were thawed and treated with RNase A (EN0531 Thermo Fisher, diluted 10×) for 1 h at 37 °C to degrade RNAs that were not packaged inside the virion. RNA was extracted using Trizol (Thermo Fisher #15596026) and resuspended in NF water. DNase (Thermo Fisher #AM1907) was used to remove the contaminant DNA. RNA was reverse transcribed (Fisher Scientific #18-080-044). The Luna qPCR (NEB #M3004) was used to quantify the generated cDNA. The OneTaq RT-PCR mix (NEB #E5315) was used to amplify the generated cDNA.

### Rapture FISH

The steps after immobilization were performed as described previously in the literature.^16^ The coverglass was washed three times with the binding buffer after virions capture. To fix virus samples, −20 °C methanol was added to samples, which were then incubated at −20 °C for 10 min. A 500 μM probe mixture was mixed with the hybridization buffer (10% dextran sulfate/10% formamide/2× SSC) and incubated with the fixed samples for 5 min. The samples were washed two times with the wash buffer (2× SSC, 10% formamide), and then 2× SSC was added for subsequent imaging.

### Image Acquisition

Microscopy was performed using a Nikon inverted research microscope eclipse Ti2-E/Ti2-E/B using a Plan Apo λ 20×/0.75 objective or Plan Apo λ 100×/1.45 oil objective. An Epi-fi LED illuminator linked to the microscope assured illumination and controlled the respective brightness of four types of LEDs of different wavelengths. Images were acquired using a Neutral Density (ND16) filter for probes coupled with Alexa 488, Alexa 594, Alexa 647, and cy3. Images were acquired and processed using ImageJ. Images acquired using the Neutral Density (ND16) filter were false-colored gray. The nuclei of live cells were stained by the NucBlue Live Cell Stain ReadyPorbes reagent (Thermo Fisher #R38605).

### Image Analysis and Quantification

After imaging, we put our data through an image analysis pipeline for semiautomated spot recognition. The pipeline, developed in Matlab, can be divided into three main steps of binarization, colocalization, and size thresholding. Briefly, in the first binarization step, we manually determined a threshold for each fluorescence channel so the number of spots can be localized well in positive control. Images of each channel shared the same thresholding level, which was applied to the images to binarize them. The binarized images were then overlapped and intersected to find the common spots between the two channels (Figures 3 and 4). Eventually, in the last step, we set a cutoff level for the particle area, in order to exclude viral aggregates from the analysis and limit the colocalized group to just individual virions (Figure S7).

## Supporting Information Available

RT-qPCR quantification of viral capture; DNA gel electrophoresis demonstrating viral genome integrity; brightfield images of cell monolayers; and detection limit for the method (PDF)

## Notes

An earlier version of the manuscript was deposited in a preprint server. Liu, Y.; Potts, J. L.; Bloch, D.; Nian, K.; McCormick, C. A., Fanari, O.; Rouhanifard, S. H. Paired Aptamer Capture and FISH Detection of Individual Virions Enables Cell-Free Determination of Infectious Titer. Posted Nov 14, 2022. DOI: 10.1101/2022.11.13.516306.
